# Supplementary material for: The Emotional Response to Pandemic of Middle- and High-School Students of an Italian Northern Province: The ERP Study
Source: Children (Basel). 2022 Jan 4;9(1):59. doi: 10.3390/children9010059 (PMC8774327; doi:10.3390/children9010059)

Supplementary Material

Table S1. Correlation matrix in adults.

	Indifference	Disbelief	Irritation	Anxiety	Aggressiveness	Concern	Fear for you	Fear for your loved ones	Loss of interest in your activities	Depression
Indifference	1									
Disbelief	0.16***	1								
Irritation	0.117**	0.361***	1							
Anxiety	-0.068	0.23***	0.338***	1						
Aggressiveness	0.165***	0.234***	0.425***	0.38***	1					
Concern	-0.067	0.22***	0.181***	0.588***	0.144***	1				
Fear for you	-0.062	0.197***	0.199***	0.651***	0.269***	0.733***	1			
Fear for your loved ones	-0.03	0.197***	0.192***	0.526***	0.11**	0.605***	0.62***	1		
Loss of interest in your activities	0.16***	0.207***	0.254***	0.275***	0.364***	0.189***	0.203***	0.19***	1	
Depression	0.015	0.179***	0.229***	0.459***	0.456***	0.308***	0.346***	0.24***	0.456***	1

*** p<0.001, ** p<0.01, * p<0.05

Dark green: correlation >0.5; light green: correlation >0.4; dark yellow: correlation >0.3; yellow: correlation >0.2; light yellow: correlation >0.1

Table S2. Correlation matrix in high school students.

	Indifference	Disbelief	Irritation	Anxiety	Aggressiveness	Concern	Fear for you	Fear for your loved ones	Loss of interest in your activities	Depression
Indifference	1									
Disbelief	0.045	1								
Irritation	0.066	0.146***	1							
Anxiety	0.096**	0.132***	0.43***	1						
Aggressiveness	0.09*	0.257***	0.536***	0.38***	1					
Concern	0.022	0.267***	0.383***	0.575***	0.408***	1				
Fear for you	-0.052	0.264***	0.306***	0.523***	0.412***	0.639***	1			
Fear for your loved ones	-0.107**	0.228***	0.254***	0.27***	0.304***	0.458***	0.477***	1		
Loss of interest in your activities	0.259***	0.073*	0.291***	0.34***	0.342***	0.274***	0.188***	0.096**	1	
Depression	0.086*	0.085*	0.388***	0.567***	0.437***	0.366***	0.368***	0.126***	0.456***	1

*** p<0.001, ** p<0.01, * p<0.05

Dark green: correlation >0.5; light green: correlation >0.4; dark yellow: correlation >0.3; yellow: correlation >0.2; light yellow: correlation >0.1

Table S3. Correlation matrix in middle school students from urban areas.

	Indifference	Disbelief	Irritation	Anxiety	Aggressiveness	Concern	Fear for you	Fear for your loved ones	Loss of interest in your activities	Depression
Indifference	1									
Disbelief	0.27*	1								
Irritation	0.034	0.044	1							
Anxiety	0.202	0.113	0.508***	1						
Aggressiveness	0.218	0.093	0.536***	0.434***	1					
Concern	0.08	0.013	0.341**	0.538***	0.157	1				
Fear for you	0.087	0.09	0.28*	0.582***	0.122	0.707***	1			
Fear for your loved ones	-0.09	0.076	0.209	0.327**	0.061	0.603***	0.524***	1		
Loss of interest in your activities	0.146	0.151	0.135	0.182	0.292*	-0.064	0.041	0.157	1	
Depression	0.031	0.016	0.533***	0.332**	0.414***	0.421***	0.306*	0.23	0.09	1

*** p<0.001, ** p<0.01, * p<0.05

Dark green: correlation >0.5; light green: correlation >0.4; dark yellow: correlation >0.3; yellow: correlation >0.2; light yellow: correlation >0.1

Table S4. Correlation matrix in middle school students from rural areas.

	Indifference	Disbelief	Irritation	Anxiety	Aggressiveness	Concern	Fear for you	Fear for your loved ones	Loss of interest in your activities	Depression
Indifference	1									
Disbelief	0.245*	1								
Irritation	0.08	0.43***	1							
Anxiety	-0.041	0.302*	0.382**	1						
Aggressiveness	-0.148	0.157	0.34**	0.46***	1					
Concern	-0.161	0.369**	0.364**	0.678***	0.469***	1				
Fear for you	-0.087	0.423***	0.394**	0.763***	0.38**	0.846***	1			
Fear for your loved ones	-0.102	0.298*	0.251*	0.445***	0.219	0.579***	0.726***	1		
Loss of interest in your activities	0.098	0.133	0.128	0.212	0.429***	0.331**	0.238	0.187	1	
Depression	-0.114	0.181	0.139	0.256*	0.345**	0.355**	0.29*	0.151	0.478***	1

*** $p<0.001$, ** $p<0.01$, * $p<0.05$

Dark green: correlation >0.5; light green: correlation >0.4; dark yellow: correlation >0.3; yellow: correlation >0.2; light yellow: correlation >0.1

Figure S1. Pairwise comparisons for the 10 items investigated in the questionnaire: adults vs. students (right radar diagram), high vs. middle school students (middle radar diagram), and middle school students from urban vs. rural areas (left radar diagram).

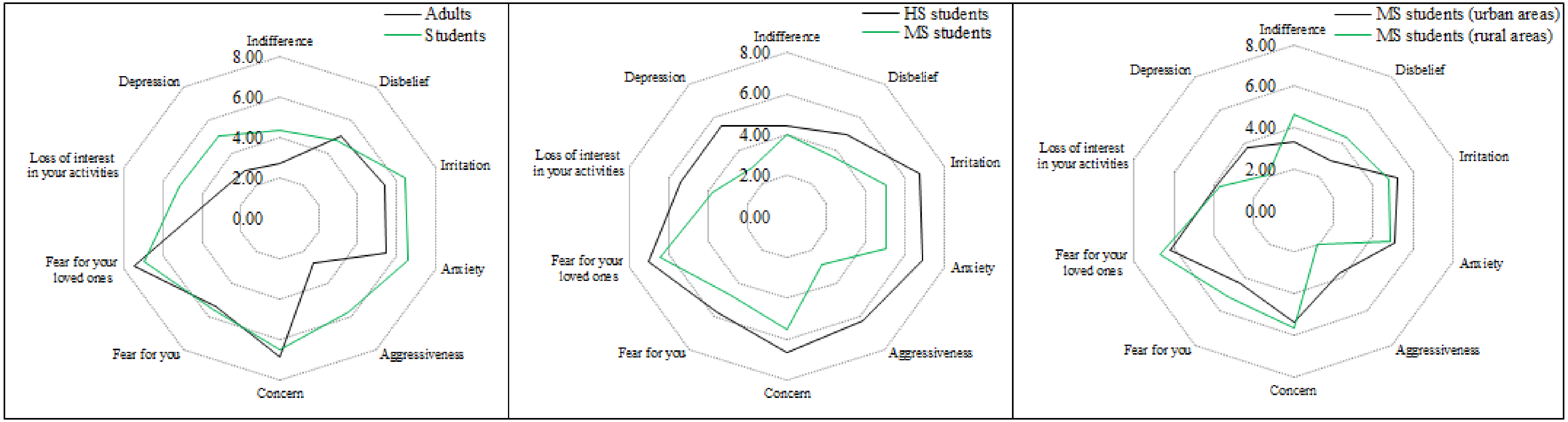

HS: high schools, MS: middle school

Figure S2. Difference among males and females regarding the 10 questions of the questionnaire in adults.

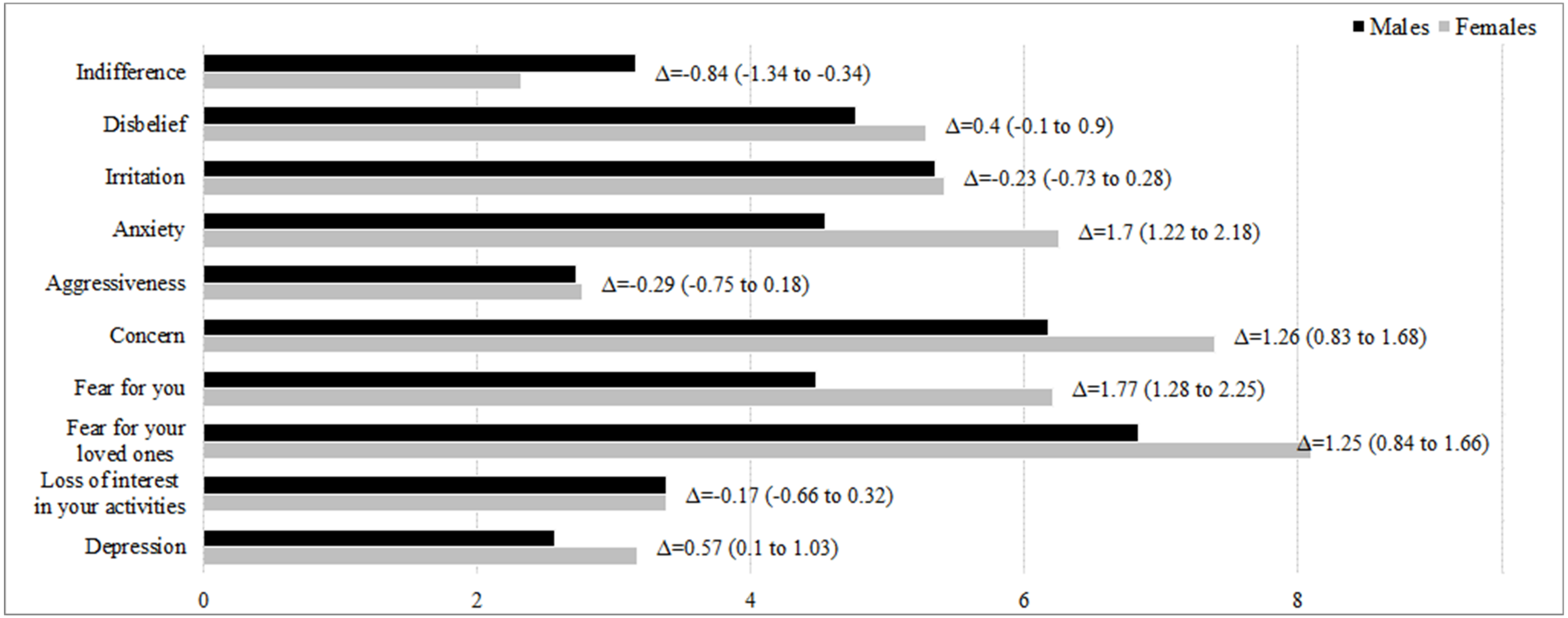

Figure S3. Difference among males and females regarding the 10 questions of the questionnaire in high school students.

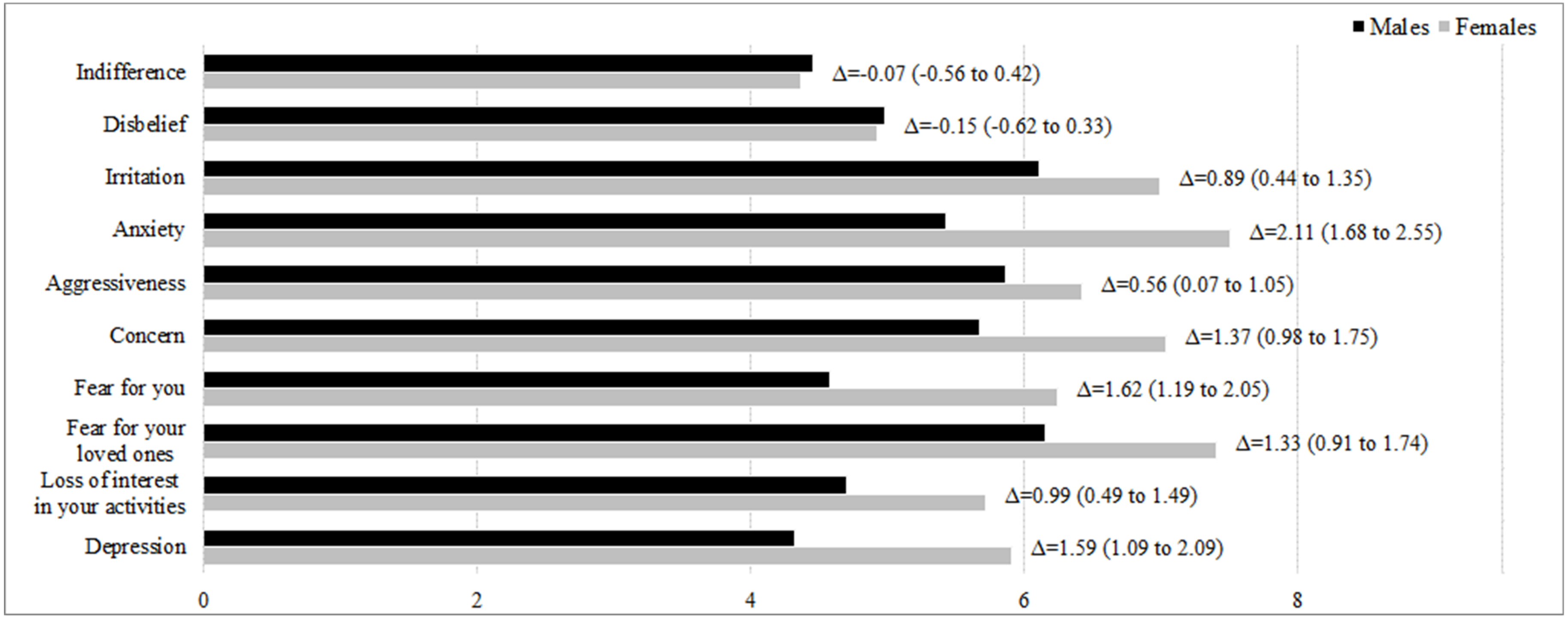

Figure S4. Difference among males and females regarding the 10 questions of the questionnaire in middle school students from urban areas.

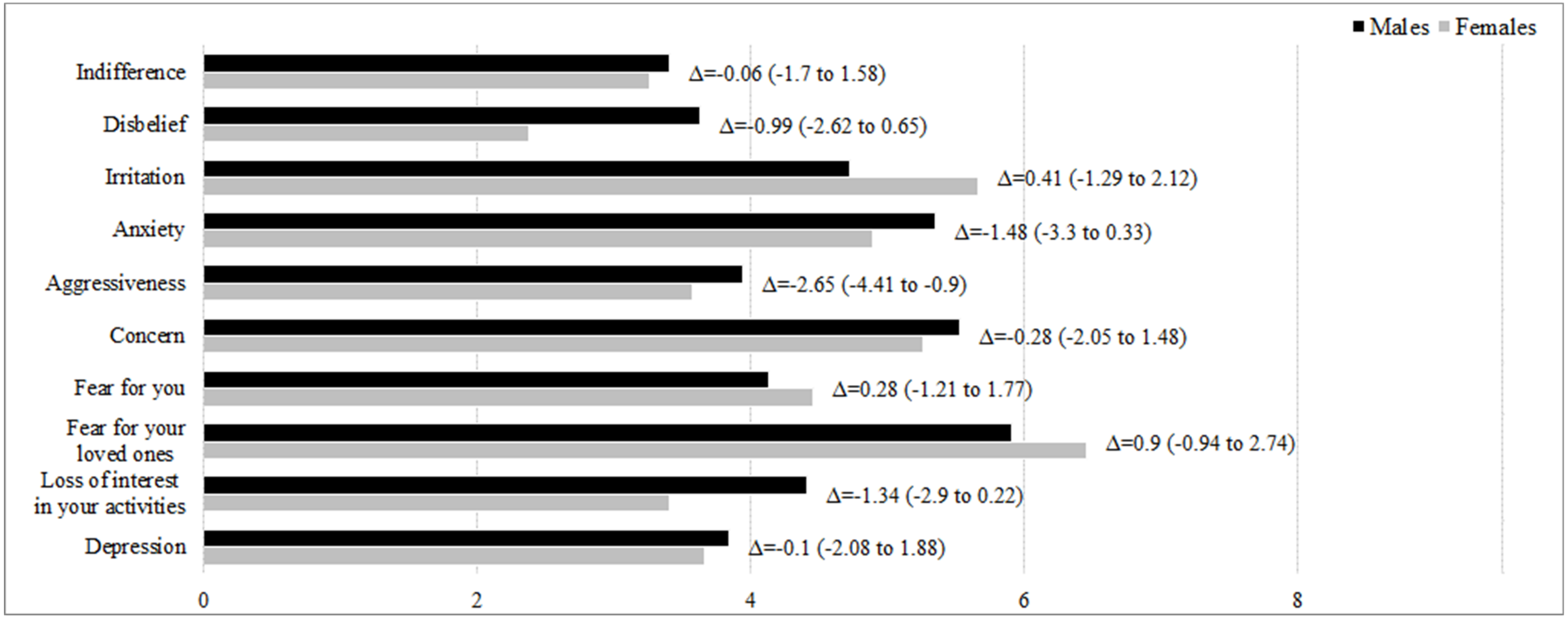

Figure S5. Difference among males and females regarding the 10 questions of the questionnaire in middle school students from rural areas.

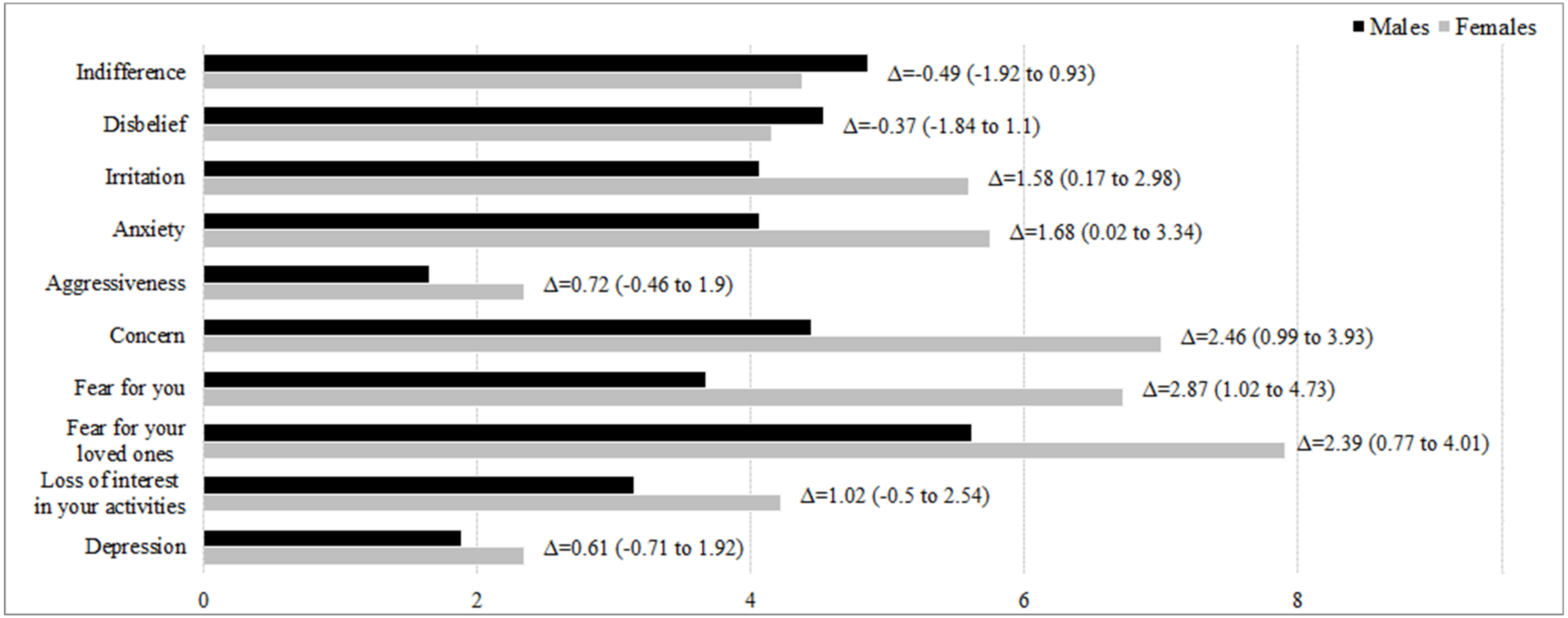

Figure S6. Correlation plots, the dimension of each bubble is proportional to the frequency of responders.

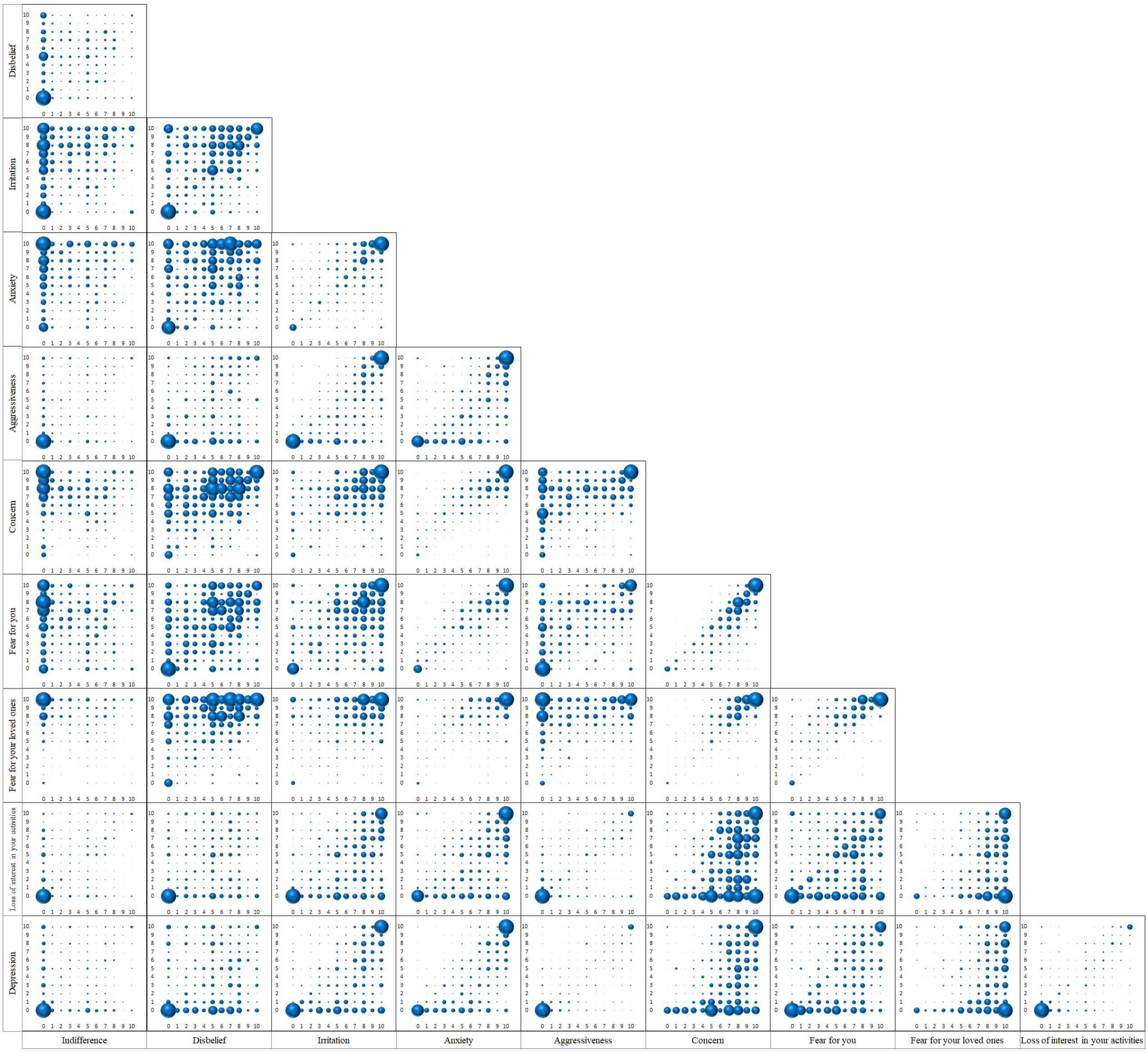

Supplement: Supplementary file 1 [file children-09-00059-s001.zip › children-1502281-supplementary.pdf]
